# Synovial changes detected by ultrasound in people with knee osteoarthritis – a meta-analysis of observational studies

**DOI:** 10.1016/j.joca.2016.03.004

**Published:** 2016-08

**Authors:** A. Sarmanova, M. Hall, J. Moses, M. Doherty, W. Zhang

**Affiliations:** † Division of Rheumatology, Orthopaedics and Dermatology, School of Medicine, University of Nottingham, UK; ‡ School of Health Sciences, University of Nottingham, UK; § Arthritis Research UK Pain Centre, Nottingham, UK

**Keywords:** Ultrasound, Synovial, Osteoarthritis, Meta-analysis, Synovitis

## Abstract

**Objectives:**

To examine the prevalence of synovial effusion, synovial hypertrophy and positive Doppler signal (DS) detected by ultrasound (US) in people with knee osteoarthritis (OA) and/or knee pain compared to that in the general population.

**Method:**

A systematic literature search was undertaken in Medline, EMBASE, Allied and Complementary Medicine, PubMed Web of Science, and SCOPUS databases in May 2015. Frequencies of US abnormalities in people with knee OA/pain, in the general population or asymptomatic controls were pooled using the random effects model. Publication bias and heterogeneity between studies were examined.

**Results:**

Twenty four studies in people with knee pain/OA and five studies of the general population or asymptomatic controls met the inclusion criteria. The pooled prevalence of US effusion, synovial hypertrophy and positive DS in people with knee OA/pain were 51.5% (95% CI 40.2 to 62.8), 41.5% (26.3–57.5) and 32.7% (8.34–63.24), respectively, which were higher than those in the general population or asymptomatic controls (19.9% (95%CI 7.8–35.3%), 14.5% (0–58.81), and 15.8 (3.08–35.36), respectively). People with knee OA (ACR criteria or radiographic OA) had greater prevalence of US abnormalities than people with knee pain (*P* = 0.037, *P* = 0.010 and *P* = 0.009, respectively).

**Conclusions:**

US detected effusion, synovial hypertrophy and DS are more common in people with knee OA/pain, compared to the general population. These abnormalities relate more to presence of OA structural changes than to pain.

## Introduction

Pathologically osteoarthritis (OA) is characterised by involvement of all joint tissues, typically with focal cartilage loss, osteophyte formation, subchondral bone remodelling, and synovial and capsular thickening. Although synovial hyperplasia in knees affected by OA is focal and less marked than in knees with rheumatoid arthritis it may still play an important role in disease pathogenesis^1^, ^2^. Cohort studies have shown a positive association between synovial pathology and disease progression^3^, ^4^, ^5^. Therefore whether synovitis is a potential biomarker of inflammatory response and therapeutic target in OA is an important research question^2^.

Ultrasound (US) imaging is used widely because it is non-invasive, has no radiation burden, is relatively inexpensive, involves a short examination time, and has good patient acceptability^6^. US detection of synovial effusion and synovial hypertrophy in knees is reported to be more sensitive than clinical examination^7^, ^8^, correlates well with histological findings^9^, ^10^ and is equivalent to MRI in visualising effusion^11^, ^12^. Strong perfusion (i.e., Doppler signal (DS)) is associated with clinical signs of inflammation (soft tissue swelling, tenderness, increased warmth) and also with histological and laboratory markers of inflammation (e.g., serum C-reactive protein) in people with inflammatory arthritis^13^, ^14^.

A number of predominantly hospital-based studies have been undertaken in knee OA to examine US detected abnormalities. However, the normal values, thresholds and frequencies of these features in the general population and in community-based people with knee pain or OA remain largely unknown. Therefore, it is of interest to systematically review studies of synovial effusion, synovial hypertrophy and positive DS in the general population and in people with knee pain or knee OA and, if possible, the prevalence and associations of such changes.

## Materials and methods

### Data sources and search strategy

Two systematic literature searches were performed using computer-based literature indexes such as Medline (1946–), EMBASE (1974–), Allied and Complementary Medicine (1985–), PubMed (1960–), Web of Science, and SCOPUS (1960–) in May, 2015. Citations and abstracts retrieved from this search were downloaded to EndNote X6.0.1 (licenced to The University of Nottingham).

The first search included (a) OA of the knee, and (b) US. The search terms were ‘‘[ultrasound or sonography or ultrasonography or doppler or dopplerography or power-doppler] and [knee osteoarthritis or knee osteoarthrosis or gonarthritis or gonarthrosis or knee pain or ((osteoarthritis or osteoarthrosis or osteophyte or joint space narrowing or degenerative joint disease(s)) and knee)]’’ (Supplementary file 1).

The second search was performed for studies that have explored prevalence of synovial changes in the general population irrespective of knee pain or knee OA using terms “[knee(s) and [ultrasound or sonography or ultrasonography or doppler or dopplerography or power-doppler] and [normal or healthy or general or population-based]” (Supplementary file 1).

### Selection criteria

Observational studies were included if they examined US-detected synovial effusion, synovial hypertrophy, or DS detected in people with knee pain/OA, in the general population or in normal/healthy controls. If studies were based on the same participants and same outcome measures, only one publication with the most detailed information was included in the review. There were no language restrictions.

Randomised controlled trials, studies in selected groups with synovial effusion or synovial hypertrophy, studies without clear definition of US-detected pathology (for example “synovitis” without description whether it is related to synovial hypertrophy or combined measure of effusion and hypertrophy), or studies not reporting the prevalence estimate were excluded as they cannot provide an adequate estimate of prevalence. Although reviews and conference proceedings were not included their references were cross-checked.

### Data extraction and outcome measures

For each included article information on authors, year of publication, study design (cross sectional, case control), population (hospital, community), sample size, age, gender, body mass index (BMI), diagnostic criteria (e.g., American College of Rheumatology (ACR)), radiographic score (e.g., Kellgren and Lawrence score (K&L)), and US findings were systematically extracted using a specifically developed data extraction form and then transferred to a database.

The primary outcome measure was frequency/prevalence of US effusion, synovial hypertrophy and DS in people with knee pain/OA and in a control or general population derived directly or indirectly from information provided in each study. The secondary outcome measure was the association of US features with OA clinical features (pain, impaired function) and radiographic structural damage. Scores for pain intensity were standardised to a common 0 (no pain) to 100 (worse pain) scale.

### Quality assessment

The Newcastle-Ottava Scales (NOS) were used for case–control and cross-sectional studies^15^ as recommended by the Cochrane Non-Randomized Studies Methods Working Group^16^. Three main criteria were assessed: participant selection and representativeness, comparability of study groups, and assessment of outcome or exposure. The quality score is based on a “star” system (range 0–9 stars for case–control studies and from 0 to 10 for cross-sectional studies) with a higher score representing better methodologic quality. The percentage of the maximum score achieved was used to present the quality of each study.

### Statistical analysis

To derive a pooled estimation of prevalence across different studies, the random effects meta-analysis was undertaken using the METAPROP package (with the Freeman–Tukey double arcsine transformation and exact binominal confidence intervals for prevalence). Heterogeneity between studies was measured using the *I*^2^ and Q test^17^, ^18^, ^19^. 95% confidence interval (CI) and *P* value of 0.05 were used for a statistically significant inference. Publication bias was assessed using funnel plots and Eggers test^20^. If the number of studies included in the meta-analysis was too small (≤4) the Harbord test was applied to measure publication bias^21^. Statistical analysis was undertaken in Stata SE V13.1 (StataCorp LP, College Station, TX, USA)^22^, ^23^.

## Results

### Selection of studies

**Fig. 1:**
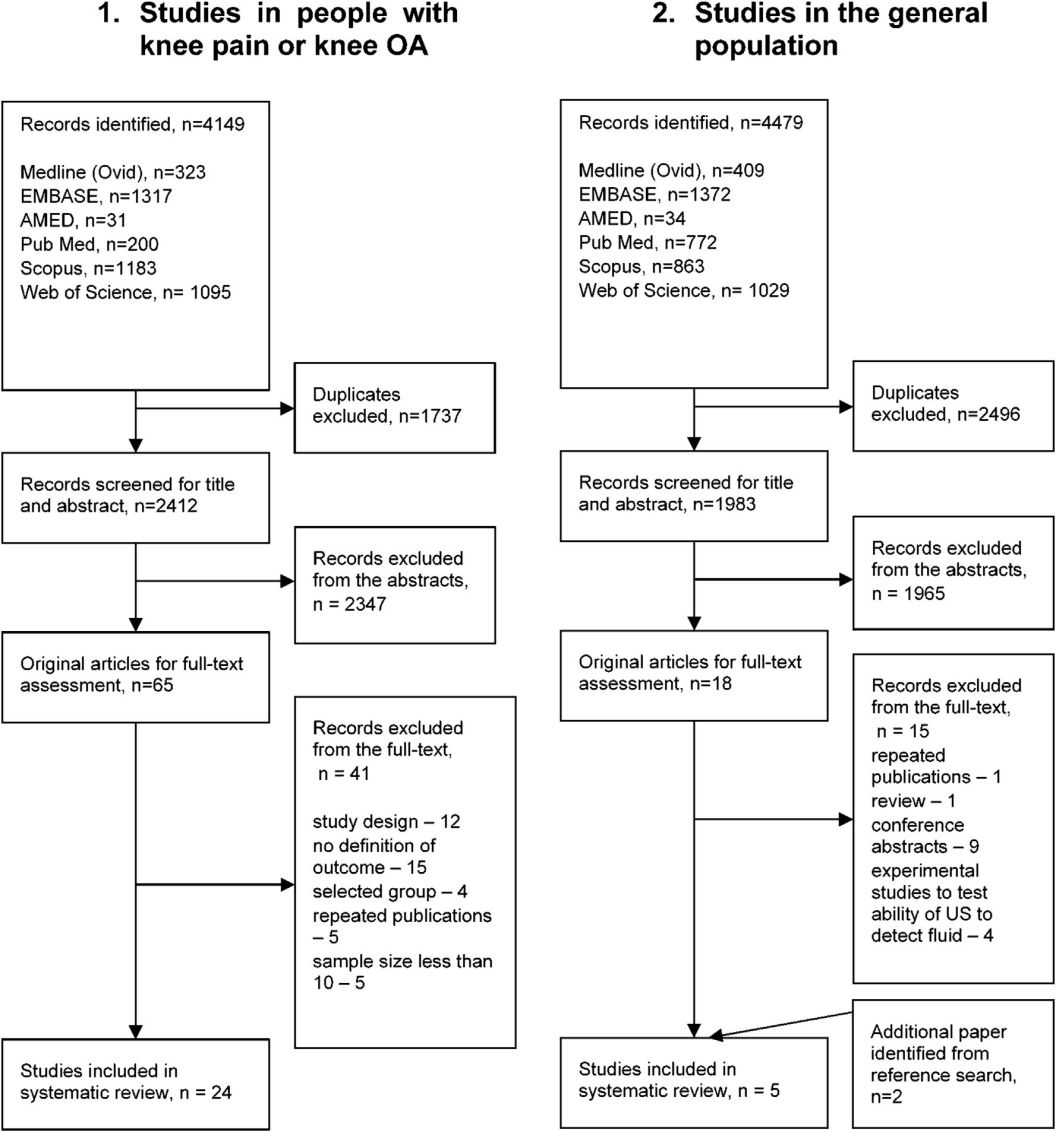
Study selections.

The first search yielded 4149 titles and abstracts, of which 65 potentially relevant publications were considered for full-text assessment. Forty-one studies were excluded by reading full-text papers, leaving a total of 24 studies which met the inclusion criteria. The second search returned 4479 citations of which only three met inclusion criteria and two additional studies were identified from the reference search (Fig. 1). All studies were published between 1990 and 2015. Three studies were translated from German, Italian and Russian^24^, ^25^, ^26^, other studies were written in English.

### Characteristics of studies

Data for prevalence were derived from both cross-sectional and case–control studies. Of 24 studies reporting the prevalence of US-detected effusion, synovial hypertrophy and DS in people with knee pain/OA, 14 were case–control and 10 were cross-sectional in design. Only four studies were community-based, the rest recruited participants from hospital populations except for four studies which did not declare the setting^11^, ^27^, ^28^, ^29^. The sample size ranged from 10 to 600 with nine studies reporting a sample size of more than 100. Age varied from 36 to 74 years. There were 20 studies of people with symptomatic knee OA (defined by ACR criteria) and four studies of people with knee pain irrespective of any underlying structural change. Three studies comprised more than one study group^30^, ^31^, ^32^.

Four cross-sectional studies and one case–control study (in comparison with rheumatoid arthritis) explored prevalence and characteristics of US features in the general population^33^, ^34^, and pain-free volunteers^24^, ^25^, ^35^. None of these five studies obtained radiographic data. Three of the five studies (range 50–488) recruited more than 100 subjects^33^, ^34^, ^35^. Age range was from 37 to 73 years.

Ten of the 29 studies were funded from academic sources, one declared no funding, one had commercial support and others did not specify funding resources. Baseline demographic characteristics (age, gender, BMI, pain assessment and radiographic score) were generally well reported. Table I summarises the main characteristics of included studies. More details are in Supplementary file 2.

**Table I:** Characteristics of the included studies

	People with knee OA/pain∗	General/normal population
Number of studies	24	5
Number of subjects	3713	1007
Mean age (years)	61.05	52.74
Women (%)	75.03	48.93
Mean BMI† (kg/m^2^)	28.2	25.33

∗ Including control groups.

† BMI – body mass index.

Definitions of US pathology varied from dichotomous measures (with different thresholds) to individual scoring systems (0–3 or 0–4 scale) or summative quantitative systems (adding effusion, synovial thickness and/or DS). Supplementary file 3 provides an overview of US scoring systems used in these studies.

### Study quality assessment

Of 24 studies in people with knee OA/pain 12 had a score of ≥50%. In cross-sectional studies the Newcastle-Ottava quality scores ranged from 2 to 9 stars with a median score of 5.5 (maximum 10). Three studies scored less than five^28^, ^36^, ^37^. In general, all samples were selected non-randomly, provided adequate definition of cases (ACR-criteria for OA diagnosis or validated tool for knee pain assessment) and blinded US assessment. The scores on each of the seven criteria and total scores for each study are presented in Supplementary file 4-1.

The quality of the case–control studies varied from 1 to 6 stars with a median score of 4 (maximum 9) (Supplementary file 4-2). Overall the majority of studies had an adequate case definition (ACR criteria or radiographic OA). The definition of controls included no history of joint disease and no OA as defined.

### Prevalence of US features in people with knee OA/pain

Of the 24 included studies, 21 had data for effusion, 13 for hypertrophy and 7 for DS. The pooled prevalence was 51.5%, 41.5% and 32.7%, respectively (Table II). Studies were highly heterogeneous, but only studies involved in the meta-analysis for hypertrophy had significant publication bias (Table II).

**Table II:** Prevalence of US-detected findings in people with knee OA/pain

	Number of studies	Number of subjects	Pooled prevalence (95% CI)	I^2^% (P_heter_)	P_pub_
**People with knee OA/pain**					
Effusion	21	3266	51.5 (40.2–62.8)	97.5 (<0.0001)	0.082∗
Synovial hypertrophy	13	1785	41.5 (26.3–57.5)	97.6 (<0.0001)	0.026∗
DS	7	538	32.7 (8.34–63.24)	98.0 (<0.0001)	0.493∗
**The general/normal population**					
Effusion	6	922	19.9 (7.81–35.34)	94.7 (<0.0001)	0.587∗
Synovial hypertrophy	4	601	14.5 (0–58.81)	98.7 (<0.0001)	0.118∗∗
DS	2	533	15.8 (3.08–35.36)	93.8 (<0.001)	–

CI: confidence interval; I^2^: inconsistency; P_heter_: p for heterogeneity; P_pub_: p for publication bias.

∗ Egger's test.

∗∗ Harbord's test.

**Fig. 2:**
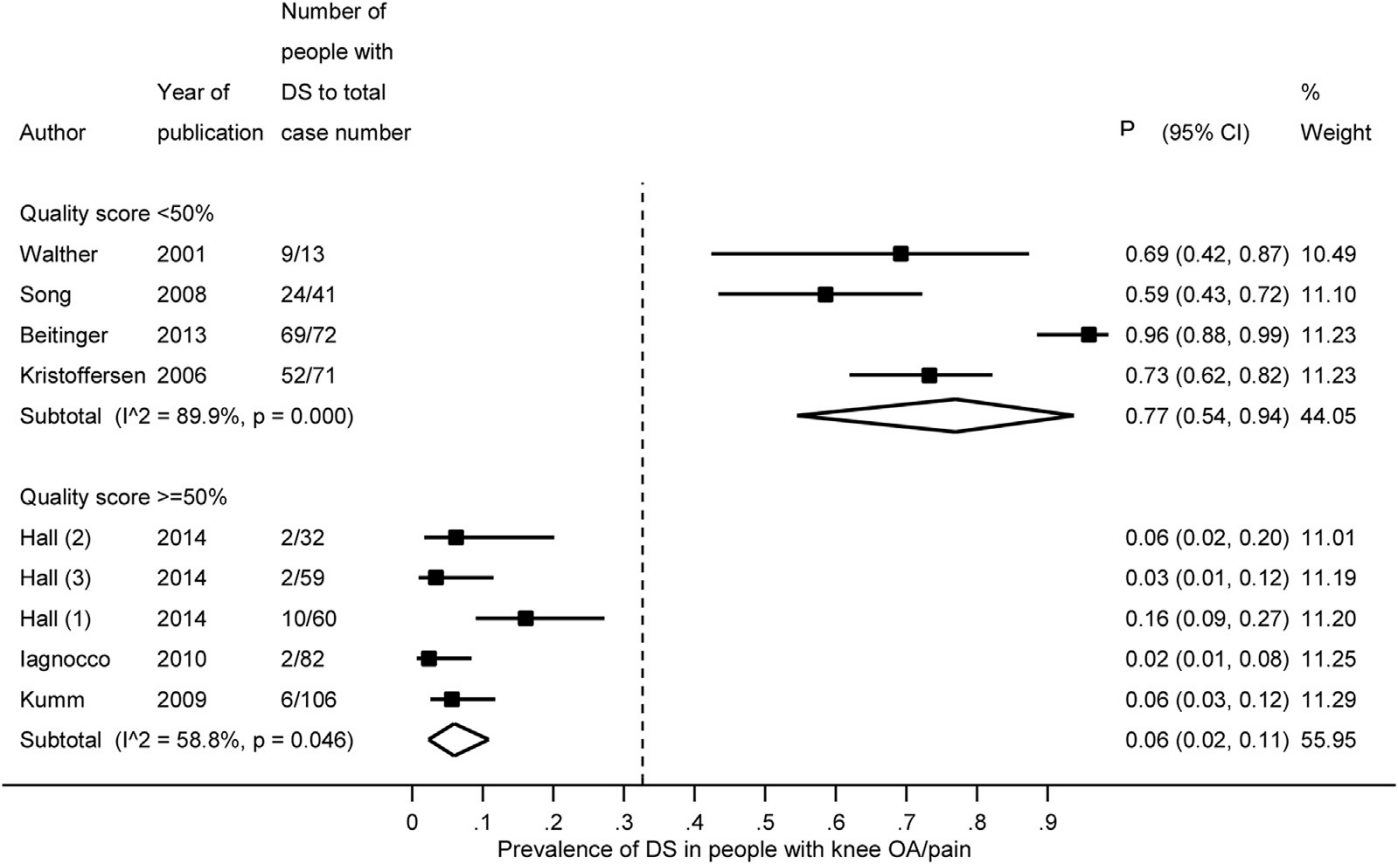
Forest plot showing the subgroup analysis by overall quality score for the prevalence of DS in people with knee OA/pain. P – prevalence rates, 95% CI – lower and upper confidence limits of the 95% confidence interval around the mean prevalence rate. The diamond in the forest plot denotes the summary prevalence and its edges the respective 95% CIs. Three groups from the study by Hall were included: (1) – people with symptomatic OA, (2) – people with radiographic OA, (3) – people with knee pain.

Several subgroup analyses were undertaken according to US threshold for abnormality, sample size of study, overall quality of study and definition of OA. The results are summarised in Table III. In general larger studies (≥100) tended to give a lower prevalence than smaller studies (<100). Similarly, higher quality studies (overall score ≥50%) tended to have a lower prevalence than lower quality studies (overall score <50%). This was especially true when DS was assessed, where a clear separation was observed between higher and lower quality studies (Fig. 2). Interestingly, people with either ACR or radiographic knee OA had greater prevalence of all three US abnormalities than people with knee pain (Table III).

**Table III:** Subgroup analysis in studies on people with knee OA/pain

Subgroup analysis	Effusion			Synovial hypertrophy			DS		
	Number of studies	Prevalence (95% CI)	P∗	Number of studies	Prevalence (95% CI)	P∗	Number of studies	Prevalence (95% CI)	P∗
**Threshold**									
≥4 mm	11	52.5 (38–66.8)		10	43.1 (26.5–60.5)				
≥2 mm	7	67.6 (55.8–78.3)		2	25.5 (14.5–38.2)				
Absent or present	6	32.7 (13.9–54.9)	0.018	3	46.9 (0–99.7)	0.234			
**Sample size**									
≥100 subjects	9	37.1 (20.8–55.2)		5	21.4 (12.4–32.1)		1		
<100 subjects	15	60.8 (48.4–72.5)	0.034	10	52.8 (29.3–75.7)	0.015	8		
**Quality score**									
<50%	10	54.7 (32.1–76.4)		5	47.1 (8.4–88.0)		4	77 (54.5–93.7)	
≥50%	14	49.2 (37.3–61.2)	0.677	10	38.5 (24.7–53.4)	0.726	5	6.0 (2.4–10.9)	<0.0001
**Case definition**									
Knee OA	14	58.7 (47–69.9)		12	49 (30.5–67.6)		7	43.8 (11.7–79.0)	
Knee pain	5	26 (5.6–54.4)	0.037	3	15.2 (3.3–33.3)	0.010	2	4.8 (1.9–8.7)	0.009
**Study design**									
Cross-sectional	11	43.0 (28.0–58.0)		5	25.0 (17.0–34.0)		2	4.0 (2.0–8.0)	
Case-control	13	59.0 (41.0–76.0)	0.180	10	50.0 (24.0–76.0)	0.07	7	44.0 (13.0–78.0)	<0.0001
**Doppler settings**									
Colour doppler							2	87.0 (80.0–92.0)	
Power doppler							7	20.0 (4.0–42.0)	
Not stated							1	6.0 (3.0–12.0)	<0.0001
**Mean age**									
≤60	9	53.0 (42.0–63.0)		5	24.0 (13.0–38.0)		1		
>60	13	61.0 (47.0–74.0)	0.070	9	55.0 (28.0–80.0)	0.050	7		
**Women proportion**									
≤70	8	51.0 (29.0–73.0)		7	31.0 (16.0–49.0)		7		
>70	14	59.0 (48.0–70.0)	0.520	7	58.0 (30.0–84.0)	0.110	1		

∗ P-test for heterogeneity between subgroups.

### Prevalence of US features in the general/normal population

Among five studies identified from the second search, two provided data on prevalence of US detected synovial effusion in the general population^33^, ^34^. In addition, four normal (i.e., asymptomatic) control groups from the case control studies^11^, ^27^, ^30^, ^38^ reported prevalence of US synovial effusion. These made a total number of six studies in this analysis (Table II). The pooled prevalence of US synovial effusion was 19.9% (95%CI 7.8–35.3%), approximately 2–3 times lower than that in people with knee OA/pain (51.5%, 95%CI 40.2–62.8%, Table II). Similarly, four studies^11^, ^27^, ^30^, ^34^ provided data for hypertrophy and two studies^30^, ^34^ for DS. The prevalence of these findings was much lower in the general/normal population than in people with knee OA/pain. The studies were highly heterogeneous but had no evidence of publication bias (Table II).

### Associations of US-detected synovial changes with pain and structural changes

Ten studies examined the relationship between knee pain and US-detected synovial changes. Overall, the most studies reported a positive association between knee effusion and pain (7 of 10 studies) and no association between synovial hypertrophy and pain (4 of 6), but there were no data for DS (Table IV).

**Table IV:** Associations between effusion and synovial hypertrophy with pain

Author, year	Sample size	Mean age (SD/range)	Proportion of women	Standardised quality score (% of the maximum score)	Association between effusion and pain	Association between synovial hypertrophy and pain
Bevers 2014∗	180	57 (9.2)	66.7	50	No association	No association
Song 2008	41	65 (6.7)	63.4	22.2	No association	No association
Ulasli 2014	86	56.2 (10.2)	80.2	60.0	No association	
Hall 2014	62	73.9 (7.8)	67.7	55.6	Positive association	Positive association
D'Agostino 2005∗	600	66.7 (9.8)	72.5	90.0	Positive association	No association
Malas 2014	61	58.88 (7.2)	83.6	40.0	Positive association	
Mendieta 2006	101	62.1 (9)	70.0	70.0	Positive association with pain on motion	
Chan 2014	193	59 (13.9)	74.1	60.0	Positive association with pain on walking, but not while sitting	Positive association with pain while sitting, but not walking
Wu 2012∗	56	62.9 (8.2)	75.0	66.7	Positive association with pain during movement, but not at rest	No association with pain on movement and at rest
Naredo 2005	50	64.3 (7.9)	88.0	55.6	Positive association with pain during movement and at rest	
% positivity					7/10	2/6

∗ Adjusted for radiographic severity.

Three studies examined knee pain on walking and at rest separately^32^, ^38^, ^39^. Two studies did not find any association between knee effusion and pain at rest^32^, ^39^, whereas this association was observed by Naredo (2005)^38^. Both studies examined synovial hypertrophy but found no association with pain on walking and indefinite results with pain at rest^32^, ^39^. Unfortunately these studies did not provide sufficient data for statistical pooling so the strength of the association between knee pain and US effusion/synovial hypertrophy remains unknown.

Only two studies examined the relationship between Doppler activity and pain, both recruited people with symptomatic knee OA with disease duration more than 6 months. Song (2009)^40^ found a positive correlation (*r* = 0.366; *P* = 0.020) between DS and knee pain in people with moderate to severe knee pain (mean pain score – 68.3 (SD 19.6)) and structural changes on radiographs (K&L ≥2). A study by Iagnocco (2010)^41^ revealed a significant association between total US score (effusion, synovial hypertrophy and DS score in both knees) and pain (*P* = 0.004) in participants with knee pain more than 20 mm on a 100 mm VAS scale (mean pain score 48.4 mm (SD 19.9).

Three studies examined the association between US-detected abnormalities and radiographic severity^30^, ^32^, ^42^. A positive association was observed in two studies which directly addressed the association between synovial changes and radiographic severity^30^, ^42^. For example, knee effusion or abnormal synovial thickness on US were associated with radiographic OA, defined as K&L ≥3 in one study with ORs of 1.91 (95% CI 1.32 to 2.77) and 2.2 (95% CI 1.33 to 3.64), respectively^42^. This association was independent of pain, whereas the association between US features and pain was highly dependent on the severity of radiographic changes and only significant in people without OA (K&L ≤2). These findings were supported by a recent study by Hall (2014)^30^, in which four groups (normal control, knee pain only, radiographic knee OA (K&L ≥2) only, and knee pain plus radiographic OA) were compared. This study found no difference between the normal control and knee pain group, but significantly higher scores in both the asymptomatic radiographic OA and symptomatic radiographic OA groups. The prevalence was 29%, 32%, 81%, and 92% for effusion (≥4 mm); 8%, 12%, 41% and 82% for hypertrophy (≥4 mm); and 2%, 3%, 6% and 16% for DS (any grade), respectively. In addition, this study followed participants for 3 months and found no association between change in pain and change in US features. The study of Wu (2012)^32^ did not explore directly the association between US findings and structural changes. Participants with knee OA who had bilateral equal K&L scores showed significant differences between symptomatic and asymptomatic knees (*P* = 0.016 for effusion and *P* < 0.001 for synovial hypertrophy), suggesting that synovial changes are related more to pain than structural severity. However this study does not allow us to draw a strong conclusion.

## Discussion

This is the first meta-analysis of US detected synovial changes in people with and without knee OA/pain. Twenty-nine observational studies including 4720 participants from different countries were included in this study. The main findings are: [1] the prevalence of US detected effusion, synovial hypertrophy and positive DS are 2–3 times higher in people with knee OA/pain than in the general population or asymptomatic control groups; and [2] the US abnormalities relate more to presence of OA structural change than to pain.

People with knee OA had significantly higher prevalence of effusion, synovial hypertrophy and DS than people with knee pain (*P* = 0.037, *P* = 0.010 and *P* = 0.009, respectively) (Table II). This may be contrary to general expectation since the three US features selected are widely considered to reflect inflammation and pain in knee OA is suggested to associate with inflammation^43^, ^44^. Importantly, however, this finding suggests that US detected synovial change (effusion, hypertrophy, DS) may mainly correlate with the degree of OA structural change and pathology, which increasingly is recognised to involve all tissues that comprise the joint, rather than represents a biomarker/mechanism that links strongly with pain production.

There was significant heterogeneity between studies with respect to prevalence of all three US features. Such heterogeneity is to be expected because a systematic review brings together studies that are diverse both clinically and methodologically (e.g., thresholds of abnormality, recruitment source, sample size, age, gender proportion, BMI, disease duration, definition of knee OA/pain). For example, among studies in people with knee OA/pain the subgroup analysis revealed that studies with quality scores lower than 50% of maximum presented significantly higher prevalence of DS (*P* < 0.0001), and studies with sample size less than 100 reported significantly higher prevalence of effusion and synovial hypertrophy (*P* = 0.034 and *P* = 0.015, respectively). This suggests that small studies tend to inflate the results – the small study effect^21^, ^45^. Care must be taken when interpreting the results from such studies as they may overestimate the prevalence of abnormalities.

The second research question was to determine relationships between US features and knee pain. The majority of studies reported a positive association between presence of effusion and knee pain (7 out of 10) but no association between synovial hypertrophy and pain (2 out of 6) (Table IV). US-detected findings were also associated with structural changes on X-ray in two of the three studies^30^, ^32^, ^42^. However, our subgroup analysis according to knee pain and knee OA suggests that these three US abnormalities relate to knee OA (either ACR symptomatic or radiographic) more than knee pain. Further study is required to explain this finding.

A paucity of information was found on the prevalence of US-detected changes in the general population and no prospective community studies were identified. Considering gender differences and possible associations between normal values and changes in the musculoskeletal system and body composition with increasing age the normal values of US detected synovial characteristics are essential for the classification and diagnosis of people with knee pain and OA. It is expected that the normal values for older adults might differ from those for younger people, since age-related changes contribute to alterations in cartilage morphology, proprioception and muscle weakness even in the absence of OA. For example, in the Framingham study the prevalence of effusion/synovitis on MRI in people without knee OA was 37% if the present/absent scale was applied but only 4% if defined by WORMS grade two or more. Such synovitis was detected significantly more often in men than women (6% and 3%, respectively; *P* = 0.02), but there was no difference in relation to presence of knee pain or BMI^46^. However, at present the characteristics of US-detected synovial abnormalities especially in older age groups remains unknown.

There are several limitations to this study. Firstly, we focused only on the knee, so the results cannot be extrapolated to other joints. Secondly, there was significant heterogeneity in the results on prevalence, so the results of this review need to be interpreted with caution. For example, differences in scanning technique were common within included studies (e.g., neutral vs flexed knee position, multi-planar vs midline scan (Supplementary file 3)) which might affect the results and together with differences in participant characteristics (age, gender, disease duration, severity of structural changes) might explain some of the between-study heterogeneity^40^, ^47^, ^48^. Thirdly, the prevalence in the general population was obtained from just a few studies including controls from case control studies. This group is neither a random sample of the general population, nor comparable to the cases with knee OA/pain. The prevalence obtained from such an assembled “normal” control group cannot be extrapolated to the prevalence in the general population.

Our study highlights the lack of information on the presence of synovial change in the knee. Although many studies have explored this question, none has investigated the distribution of these features in the general population, hence the threshold for abnormality has yet to be established. US-detected pathology should be described in detail and studies should provide sufficient information on definition and thresholds used. The heterogeneity across studies highlights the need for a standard protocol in order to allow comparability between studies in the future.

In conclusion, US detected effusion, synovial hypertrophy and DS are more common in people with knee OA/pain, compared to the general population. These abnormalities relate more to presence of OA structural changes than to pain. Further studies on the reasons of this difference, normal values of the US features and their thresholds of abnormalities are warranted.

## Contributions

Study concept and design: WZ, MD and AS. The first review (AS) ran the literature search, screened relevant full-text articles for inclusion criteria, extracted data for all studies included in the review, and assessed the study quality. MH and JM (for the1st and 2nd search, respectively) reviewed included articles, extracted data and performed the quality assessment independently from the first reviewer (AS). Any inconsistencies were solved by consensus with involvement of a further experienced reviewer (WZ). AS analysed data and wrote the first draft. All authors (AS, MH, JM, MD and WZ) contributed to data interpretation and editing of final paper. All authors approved the final version for publication.

## Competing interests

None.

## Role of the funding source

The sponsors of the study had no role in design and conduct of the study; collection, management, analysis and interpretation of the data; and preparation, review or approval of the manuscript.
