# Supplementary material for: Synovial changes detected by ultrasound in people with knee osteoarthritis – a meta-analysis of observational studies
Source: Osteoarthritis Cartilage. 2016 Aug;24(8):1376–83. doi: 10.1016/j.joca.2016.03.004 (PMC4967443; doi:10.1016/j.joca.2016.03.004)
Supplement: Supplementary file 1 [file mmc1.docx]

Supplementary file 1: Search strategy for systematic literature review.

| Database | Search strategy | Articles identified |
| --- | --- | --- |
|  | **Systematic search I (December 2014):** |  |
| Ovid MEDLINE | 1 knee osteoarthritis.mp. or exp Knee Osteoarthritis/ [mp=title, abstract, original title, name of substance word, subject heading word, keyword heading word, protocol supplementary concept word, rare disease supplementary concept word, unique identifier]  2 knee osteoarthrosis.mp.  3 (gonarthritis or gonarthrosis).mp. [mp=title, abstract, original title, name of substance word, subject heading word, keyword heading word, protocol supplementary concept word, rare disease supplementary concept word, unique identifier]  4 knee pain.mp. or exp Knee Pain/ [mp=title, abstract, original title, name of substance word, subject heading word, keyword heading word, protocol supplementary concept word, rare disease supplementary concept word, unique identifier]  5 osteoarthritis.mp. or exp OSTEOARTHRITIS/ [mp=title, abstract, original title, name of substance word, subject heading word, keyword heading word, protocol supplementary concept word, rare disease supplementary concept word, unique identifier]  6 osteoarthrosis.mp.  7 osteophyte.mp. or exp OSTEOPHYTE/ [mp=title, abstract, original title, name of substance word, subject heading word, keyword heading word, protocol supplementary concept word, rare disease supplementary concept word, unique identifier]  8 joint space narrowing.mp.  9 degenerative joint disease$.mp.  10 5 or 6 or 7 or 8 or 9  11 knee.mp. or exp KNEE/ [mp=title, abstract, original title, name of substance word, subject heading word, keyword heading word, protocol supplementary concept word, rare disease supplementary concept word, unique identifier]  12 10 and 11  13 1 or 2 or 3 or 4 or 12  14 ultrasound.mp.  15 sonography.mp. or exp ultrasonography/ [mp=title, abstract, original title, name of substance word, subject heading word, keyword heading word, protocol supplementary concept word, rare disease supplementary concept word, unique identifier]  16 doppler.mp. or exp dopplerography/ [mp=title, abstract, original title, name of substance word, subject heading word, keyword heading word, protocol supplementary concept word, rare disease supplementary concept word, unique identifier]  17 Ultrasonography, Doppler, Color/ or Ultrasonography, Doppler/ or powerdoppler. mp.  18 14 or 15 or 16 or 17  19 13 and 18  20 limit 19 to humans | 323 |
| Embase | 1 knee osteoarthritis.mp. or exp Knee Osteoarthritis/ [mp=title, abstract, subject headings, heading word, drug trade name, original title, device manufacturer, drug manufacturer, device trade name, keyword]  2 knee osteoarthrosis.mp.  3 (gonarthritis or gonarthrosis).mp. [mp=title, abstract, subject headings, heading word, drug trade name, original title, device manufacturer, drug manufacturer, device trade name, keyword]  4 knee pain.mp. or exp Knee Pain/ [mp=title, abstract, subject headings, heading word, drug trade name, original title, device manufacturer, drug manufacturer, device trade name, keyword]  5 osteoarthritis.mp. or exp OSTEOARTHRITIS/ [mp=title, abstract, subject headings, heading word, drug trade name, original title, device manufacturer, drug manufacturer, device trade name, keyword]  6 osteoarthrosis.mp.  7 osteophyte.mp. or exp OSTEOPHYTE/ [mp=title, abstract, subject headings, heading word, drug trade name, original title, device manufacturer, drug manufacturer, device trade name, keyword]  8 joint space narrowing.mp.  9 degenerative joint disease$.mp.  10 5 or 6 or 7 or 8 or 9  11 knee.mp. or exp KNEE/ [mp=title, abstract, subject headings, heading word, drug trade name, original title, device manufacturer, drug manufacturer, device trade name, keyword]  12 10 and 11  13 1 or 2 or 3 or 4 or 12  14 ultrasound.mp.  15 sonography.mp. or exp ultrasonography/ [mp=title, abstract, subject headings, heading word, drug trade name, original title, device manufacturer, drug manufacturer, device trade name, keyword]  16 doppler.mp. or exp dopplerography/ [mp=title, abstract, subject headings, heading word, drug trade name, original title, device manufacturer, drug manufacturer, device trade name, keyword]  17 Ultrasonography, Doppler, Color/ or Ultrasonography, Doppler/ or powerdoppler. mp.  18 14 or 15 or 16 or 17  19 13 and 18  20 limit 19 to humans | 1317 |
| AMED | 1 knee osteoarthritis.mp. or exp Knee Osteoarthritis/ [mp=abstract, heading words, title]  2 knee osteoarthrosis.mp.  3 (gonarthritis or gonarthrosis).mp. [mp=abstract, heading words, title]  4 knee pain.mp. or exp Knee Pain/ [mp=abstract, heading words, title]  5 osteoarthritis.mp. or exp OSTEOARTHRITIS/ [mp=abstract, heading words, title]  6 osteoarthrosis.mp.  7 osteophyte.mp. or exp OSTEOPHYTE/ [mp=abstract, heading words, title]  8 joint space narrowing.mp.  9 degenerative joint disease$.mp.  10 5 or 6 or 7 or 8 or 9  11 knee.mp. or exp KNEE/ [mp=abstract, heading words, title]  12 10 and 11  13 1 or 2 or 3 or 4 or 12  14 ultrasound.mp.  15 sonography.mp. or exp ultrasonography/ [mp=abstract, heading words, title]  16 doppler.mp. or exp dopplerography/ [mp=abstract, heading words, title]  17 Ultrasonography, Doppler, Color/ or Ultrasonography, Doppler/ or powerdoppler.  mp.  18 14 or 15 or 16 or 17  19 13 and 18 | 31 |
| PubMed | ((knee[Title/Abstract] AND ((((osteoarthritis[Title/Abstract] OR osteoarthrosis[Title/Abstract]) OR osteophyte$ [Title/Abstract]) OR joint space narrowing[Title/Abstract]) OR degenerative joint disease$ [Title/Abstract])) OR ((((knee osteoarthritis[Title/Abstract] OR (exp[All Fields] AND ("osteoarthritis, knee"[MeSH Terms] OR ("osteoarthritis"[All Fields] AND "knee"[All Fields]) OR "knee osteoarthritis"[All Fields] OR ("knee"[All Fields] AND "osteoarthritis"[All Fields])))) AND Title/Abstract[All Fields] OR ("osteoarthritis, knee"[MeSH Terms] OR ("osteoarthritis"[All Fields] AND "knee"[All Fields]) OR "knee osteoarthritis"[All Fields] OR ("knee"[All Fields] AND "osteoarthrosis"[All Fields]) OR "knee osteoarthrosis" [All Fields])) AND Title/Abstract[All Fields] OR (gonarthritis[Title/Abstract] OR ("osteoarthritis, knee"[MeSH Terms] OR ("osteoarthritis"[All Fields] AND "knee"[All Fields]) OR "knee osteoarthritis"[All Fields] OR "gonarthrosis"[All Fields]))) AND Title/Abstract[All Fields] OR (knee pain[Title/Abstract] OR (exp[All Fields] AND ("knee"[MeSH Terms] OR "knee"[All Fields] OR "knee joint"[MeSH Terms] OR ("knee"[All Fields] AND "joint"[All Fields]) OR "knee joint"[All Fields]) AND ("pain"[MeSH Terms] OR "pain"[All Fields]))) AND Title/Abstract[All Fields])) AND ((ultrasound[Title/Abstract] OR sonography[Title/Abstract]) OR ultrasonography [Title/Abstract]) | 200 |
| Scopus | knee osteoarthritis or Knee Osteoarthritis OR knee osteoarthrosis OR (gonarthritis or gonarthrosis) OR knee pain or Knee Pain OR ((osteoarthritis or OSTEOARTHRITIS OR osteoarthrosis OR osteophyte or OSTEOPHYTE OR joint space narrowing OR degenerative joint disease) AND knee or KNEE) AND ultrasound OR sonography or ultrasonography OR doppler or dopplerography OR Ultrasonography, Doppler, Color or Ultrasonography, Doppler or power-doppler | 1183 |
| Web of science | TITLE-ABS-KEY(((knee osteoarthritis) OR (knee osteoarthrosis) OR (gonarthritis or gonarthrosis) OR (knee pain) OR (((osteoarthritis) OR (osteoarthrosis) OR (osteophyte) OR (joint space narrowing) OR (degenerative joint disease$)) and (knee))) AND ((ultrasound) OR (ultrasonography) OR (doppler) OR (dopplerography) OR (power-doppler) OR (color doppler)) ) AND ( LIMIT-TO(DOCTYPE,"ar" ) OR LIMIT-TO(DOCTYPE,"re" ) OR LIMIT-TO(DOCTYPE,"cp" ) OR LIMIT-TO(DOCTYPE,"le" ) ) AND ( LIMIT-TO(SUBJAREA,"MEDI" ) OR LIMIT-TO(SUBJAREA,"HEAL" ) ) AND ( LIMIT-TO (EXACTKEYWORD, "Human" ) OR LIMIT-TO(EXACTKEYWORD,"Humans" ) ) AND ( EXCLUDE(SUBJAREA,"BIOC" ) OR EXCLUDE(SUBJAREA,"NEUR" ) OR EXCLUDE(SUBJAREA,"ENGI" ) OR EXCLUDE(SUBJAREA,"PHYS" ) OR EXCLUDE(SUBJAREA,"AGRI" ) ) | 1095 |
|  | **Systematic search II (May 2015):** |  |
| Ovid 1 MEDLINE | 1 Knee/  2 knee$.mp. [mp=title, abstract, original title, name of substance word, subject heading word, keyword heading word, protocol supplementary concept word, rare disease supplementary concept word, unique identifier]  3 ultrasound.mp.  4 Ultrasonography, Doppler, Color/ or sonography.mp. or Ultrasonography/  5 Ultrasonography, Doppler, Color/ or Ultrasonography, Doppler, Pulsed/ or Ultrasonography, Doppler, Duplex/ or doppler*.mp. or Ultrasonography, Doppler/  6 normal.mp.  7 healthy.mp.  8 general.mp.  9 population-based.mp.  10 1 or 2  11 3 or 4 or 5  12 6 or 7 or 8 or 9  13 10 and 11 and 12  14 limit 13 to (humans and "all adult (19 plus years)") | 409 |
|  |  |  |
| Embase | 1 Knee/  2 knee$.mp. [mp=title, abstract, original title, name of substance word, subject heading word, keyword heading word, protocol supplementary concept word, rare disease supplementary concept word, unique identifier]  3 ultrasound.mp.  4 Ultrasonography, Doppler, Color/ or sonography.mp. or Ultrasonography/  5 Ultrasonography, Doppler, Color/ or Ultrasonography, Doppler, Pulsed/ or Ultrasonography, Doppler, Duplex/ or doppler*.mp. or Ultrasonography, Doppler/  6 normal.mp.  7 healthy.mp.  8 general.mp.  9 population-based.mp.  10 1 or 2  11 3 or 4 or 5  12 6 or 7 or 8 or 9  13 10 and 11 and 12  14 limit 13 to (humans and "all adult (19 plus years)")  limit 14 to human | 1372 |
|  |  |  |
| AMED | 1 Knee/  2 knee$.mp. [mp=title, abstract, original title, name of substance word, subject heading word, keyword heading word, protocol supplementary concept word, rare disease supplementary concept word, unique identifier]  3 ultrasound.mp.  4 Ultrasonography, Doppler, Color/ or sonography.mp. or Ultrasonography/  5 Ultrasonography, Doppler, Color/ or Ultrasonography, Doppler, Pulsed/ or Ultrasonography, Doppler, Duplex/ or doppler*.mp. or Ultrasonography, Doppler/  6 normal.mp.  7 healthy.mp.  8 general.mp.  9 population-based.mp.  10 1 or 2  11 3 or 4 or 5  12 6 or 7 or 8 or 9  13 10 and 11 and 12  14 limit 13 to (humans and "all adult (19 plus years)")  limit 14 to human | 34 |
| PubMed | ((((knee) OR knee$)) AND ((((((ultrasound) OR sonography) OR ultrasonography) OR doppler) OR dopplerography) OR power-doppler)) AND (((healthy) OR general) OR normal) limit to humans | 772 |
| Scopus | TITLE-ABS-KEY(("knee" OR "knee$") AND ("ultrasound" OR "sonography" OR "ultrasonography" OR "doppler" OR "dopplerography" OR "power-doppler") AND ("healthy" OR "general" OR "normal")) limit to human, articles  TITLE-ABS-KEY  (("knee" OR "knee$") AND ("ultrasound" OR "sonography" OR "ultrasonography" OR "doppler" OR "dopplerography" OR "power-doppler") AND ("healthy" OR "general" OR "normal")) AND (LIMIT-TO (DOCTYPE, "ar") OR LIMIT-TO (DOCTYPE,"re") OR LIMIT-TO (DOCTYPE,"cp") OR LIMIT-TO (DOCTYPE,"ip")) AND (LIMIT-TO(EXACTKEYWORD, "Human")) | 863 |
| Web of science | # 1 TS=(knee OR knee*) Indexes=SCI-EXPANDED, SSCI, A&HCI, CPCI-S, CPCI-SSH, BKCI-S, BKCI-SSH, CCR-EXPANDED, IC Timespan=All years  # 2 TS=(ultrasound OR sonography OR ultrasonography OR doppler OR dopplerography OR power-doppler) Indexes=SCI-EXPANDED, SSCI, A&HCI, CPCI-S, CPCI-SSH, BKCI-S, BKCI-SSH, CCR-EXPANDED, IC Timespan=All years  # 3 TS=(normal OR healthy OR general OR population-based) Indexes=SCI-EXPANDED, SSCI, A&HCI, CPCI-S, CPCI-SSH, BKCI-S, BKCI-SSH, CCR-EXPANDED, IC Timespan=All years  # 4 (#3 AND #2 AND #1) Indexes=SCI-EXPANDED, SSCI, A&HCI, CPCI-S, CPCI-SSH, BKCI-S, BKCI-SSH, CCR-EXPANDED, IC Timespan=All years | 1029 |
