# Supplementary material for: Synovial changes detected by ultrasound in people with knee osteoarthritis – a meta-analysis of observational studies
Source: Osteoarthritis Cartilage. 2016 Aug;24(8):1376–83. doi: 10.1016/j.joca.2016.03.004 (PMC4967443; doi:10.1016/j.joca.2016.03.004)
Supplement: Supplementary file 2 [file mmc2.docx]

Supplementary file 2: Summary on prevalence of effusion, synovial hypertrophy and DS in people with knee pain and OA and its association with pain

| **Author, year** | **Source of population** | | **Group characteristics** | | **Sample size, n** | | **Mean age (SD/**  **range)** | **Proportion of women** | | **Proportion of people with K&L≥2** | | **Mean pain score (SD)** | | | **Knee effusion** | | | | | **Synovial hypertrophy** | | | | **Prevalence of DS, %** |
| --- | --- | --- | --- | --- | --- | --- | --- | --- | --- | --- | --- | --- | --- | --- | --- | --- | --- | --- | --- | --- | --- | --- | --- | --- |
|  |  |  |  |  |  |  |  |  |  |  |  |  |  |  | **Threshold in mm** | | **Prevalence, %** | | **Depth in mm, mean (SD)** | **Threshold in mm** | **Prevalence, %** | | **Depth in mm, mean (SD)** |  |
| **Cross-sectional studies** | | | | |  |  | | |  | |  | |  |  | |  | |  | |  |  |  | | |
| **Knee pain** | |  | |  |  |  | | |  | |  | |  |  | |  | |  | |  |  |  | | |
| Kumm, 2009 | community | | duration ≥3 month | | 106 | | 49 (36-58) | 68.87 | | 12.26 | |  | | | 4 | | 25.47 | |  | 4 | 31.13 | |  | 5.66 |
| Picerno, 2013 | hospital | | >18 years old | | 399 | | 56.2 (16.3) | 74.94 | |  | |  | | | 2 | | 62.91 | |  |  |  | |  |  |
| Arthul, 2014 | hospital | |  | | 276 | |  |  | |  | |  | | | +/-* | | 7.97 | |  |  |  | |  |  |
| **Symptomatic OA** | |  | |  |  |  | | |  | |  | |  |  | |  | |  | |  |  | |  | |
| Bevers, 2014 | hospital | |  | | 180 | | 57 (9.2) | 66.67 | | 60 | | 61 (17) | | | +/-* | | 10.91 | |  | 2 | 20.56 | |  |  |
| Chan, 2014 | community | | >40 years old | | 193 | | 59 (13.9) | 74.09 | | 61.14 | | 56 (25) | | | 4 | | 32.2 | | 6.73 (4.3) | 4 | 37.82 | | 4.36 (2.2) |  |
| D'Agostino, 2005 | hospital | | >18 years old, duration >6 month, K&L 1-4, pain last 48 hours ≥30 mm | | 600 | | 66.7 (9.8) | 72.5 | |  | | 63 (18.5) | | | 4 | | 43.5 | | 4.0 (4.6) | 4 | 16.67 | | 2.1 (2.5) |  |
| Ulasli, 2014 | hospital | | >30 years old | | 86 | | 56.2 (10.2) | 80.23 | |  | | 65.2 (11.7) | | | 4 | | 73.84 | | 4.38** |  |  | |  |  |
| Mendieta 2006 | hospital | | pain≥30 mm | | 81 | | 66.75 (8.67) | 96.3 | | 93.83 | |  | | | 2 | | 79.01 | |  |  |  | |  |  |
| Malas, 2014 | not declared | |  | | 61 | | 58.88 (7.2) | 83.61 | | - | |  | | | +/-* | | 29.51 | |  |  |  | |  |  |
| Iagnocco, 2014 | hospital | | duration>6 month, pain ≥20 mm | | 82 | | 63.2 (8.1) | 64.63 | | - | | 48.4 (19.9) | | | +/-* | | 42.68 | |  | +/-* | 21.95 | | - | 2.44 |
| **Radiographic OA** | |  | |  |  |  | | |  | |  | |  |  | |  | |  | |  |  | |  | |
| Mendieta 2006 | hospital | |  | | 20 | | 62.1 (9) | 70 | | 65 | |  | | | 2 | | 35 | |  |  |  | |  |  |
| **Case-control studies** | | | | |  |  | | |  | |  | |  |  | |  | |  | |  |  | |  | |
| **Knee pain** | | | | |  |  | | |  | |  | |  |  | |  | |  | |  |  | |  | |
| Blankstein, 2006 | not declared | |  | | 110 | | 51 (35-68) | - | | - | |  | | | +/-* | | 10.91 | |  | +/-* | 6.36 | |  |  |
| Hall 2014 | community | |  | | 59 | | 63.8 (8.8) | 55.93 | | - | | 48.9 (22) | | | 4 | | 32.2 | | 3.4 (3.2) | 4 | 11.86 | | 1.0 (1.9) | 3.39 |
| **Symptomatic OA** | | | | |  |  | | |  | |  | |  |  | |  | |  | |  |  | |  | |
| Hall, 2014 | community | | K&L≥2, pain≥30mm | | 62 | | 73.9 (7.8) | 67.74 | | 100 | | 48.2 (24.6) | | | 4 | | 91.94 | | 8.1 (4.0) | 4 | 82.26 | | 6.7 (3.3) | 16.13 |
| Naredo, 2005 | hospital | |  | | 50 | | 64.3 (7.9) | 88 | | - | |  | | | 4 | | 42 | | - |  |  | |  |  |
| Zivanovic, 2009 | military academy | | duration>6 month | | 88 | | 69.97 (9.37) | 77.27 | | - | |  | | | 4 | | 75 | | 11.38 (4.44) | 4 | 67.05 | | 6.09 (2.8) |  |
| Wu, 2012 | hospital | | equal K&L in both knees, pain ≥40 mm | | 56 | | 62.9 (8.2) | 75 | | 98.21 | |  | | | 4 | | 27.68 | | - | 4 | 83.93 | | - |  |
| Tarhan, 2003 | not declared | | >45 years old, pain within 4 weeks | | 58 | | 57.4 (8.5) | 82.76 | | - | | 58.9 (20.2) | | | 2 | | 67.24 | | - | 2 | 32.76 | | - |  |
| Chatzopoloulos, 2008 | hospital | | consulted for radiation synovectomy | | 196 | | 69 (49-87) | 74.49 | | - | |  | | | 2 | | 84.69 | | - |  |  | |  |  |
| Jung, 2006 | hospital | |  | | 51 | | 62 | 96.08 | | - | | 50 | | | 2 | | 49.02 | | - |  |  | |  |  |
| Song, 2009 | hospital | | K&L≥2, duration>6 month, pain ≥40mm | | 41 | | 65 (6.7) | 63.41 | | 100 | | 68.3 (19.6) | | | 2 | | 78.05 | | 6.1 (2.8) | 4 | 17.07 | | 1.9 (1.9) | 58.54 |
| Svetlova, 2010 | hospital | | duration 2-36 month, K&L 0-2 | | 308 | | 46.2 (11.5) | 78.25 | | 30.52 | | 60.13 (15.62) | | | +/-* | | 29.55 | |  |  |  | |  |  |
| Kristoffersen, 2006 | community | | >50 years old, pain ≥40 mm, osteophytes on x-ray | | 71 | | 68 (35-88) | 76.06 | | - | |  | | | +/-* | | 85.92 | | - | +/-* | 100 | |  | 73.24 |
| Tchetina, 2013 | hospital | | postmenopausal women, K&L≥2, pain ≥40 mm | | 47 | |  | 100 | | 100 | | (40-70) | | |  | |  | |  | 4 | 48.94 | |  |  |
| Beitinger, 2013 | hospital | | scheduled for TKR | | 72 | | 68 | - | |  | |  | | |  | |  | |  |  |  | |  | 95.83 |
| Walther, 2001 | hospital | | scheduled for TKR | | 13 | | 72.1 | 61.54 | |  | |  | | |  | |  | |  |  |  | |  | 69.23 |
| **Radiographic OA** | |  | |  |  |  | | |  | |  | |  |  | |  | |  | |  |  | |  | |
| Hall, 2014 | community | |  | | 32 | | 73.1 (7.9) | 59.38 | | 100 | | 7.2 (14.4) | | | 4 | | 81.25 | | 6 (2.8) | 4 | 40.63 | | 3.9 (3.9) | 6.25 |
|  |  | |  | |  | |  |  | |  | |  | | |  | |  | |  |  |  | |  |  |
| **General/normal population** | | | | |  | |  |  | |  | |  | | |  | |  | |  |  |  | |  |  |
| Abraham, 2014 | community | | population-based (the Newcastle thousand families birth cohort) | | 311 | | 63  (61-63) | 55.31 | |  | |  | | | 4 | | 24 | |  |  |  | |  |  |
| D'Agostino, 2015 | community | | population-based (the Bruneck cohort) | | 488 | | 72.57 (8.53) | 53.48 | |  | |  | | | +/-*** | | 60.4 | |  | +/-*** | 66.59 | |  | 24.83 |
| Martino, 1992 | not declared | | healthy volunteers | | 50 | | 37  (14-58 | 34 | |  | |  | | |  | |  | | 2.6 (1-4 mm) (effusion plus thickening) |  |  | |  |  |
| Mielke, 1990 | not declared | | healthy volunteers | | 56 | | (21-75) |  | |  | |  | | |  | |  | | 2.0 (0.4) |  |  | | 1.7 (0.3) |  |
| Schmidt, 2004 | not declared | | white volunteers | | 102 | | 38.4  (20-60) | 52.94 | |  | |  | | |  | |  | | 2.4 (1.25) |  |  | | 2.4 (1.2) |  |
| **Control groups** | | | | |  | |  |  | |  | |  | | |  | |  | |  |  |  | |  |  |
| Hall, 2014 | community | | people recruited from community-based studies (No pain, no X-ray changes) | | 90 | | 71 (7.9) | 70 | | 0 | | 6.6 (11.0) | | | 4 | | 28.88 | | 2.6 (2.7) | 4 | 7.78 | | 0.7 (1.5) | 2.22 |
| Naredo, 2005 | hospital | | Healthy without knee pain (X-ray assessment was not reported) | | 10 | | 68 (9.4) | 80 | |  | |  | | | 4 | | 0 | | 2.3 (0.7) |  |  | |  |  |
| Tarhan, 2003 | not declared | | Healthy without knee pain (X-ray assessment was not reported) | | 16 | | 59.1 (9.8) | 75 | |  | |  | | | 2 | | 16.13 | |  | 2 | 0 | |  |  |
| Beitinger, 2013 | hospital | | healthy volunteers (age, sex- adjusted) | | 52 | |  |  | |  | |  | | | +/-*** | | 5.77 | |  | +/-*** | 3.85 | |  |  |

Notes: * - reported prevalence as absent or present

** - SD was not provided

*** - reported prevalence as absent or present.

Abbreviations: DS – Doppler signal; K&L - Kellgren and Lawrence; SD- standard deviation.
