# Supplementary material for: Synovial changes detected by ultrasound in people with knee osteoarthritis – a meta-analysis of observational studies
Source: Osteoarthritis Cartilage. 2016 Aug;24(8):1376–83. doi: 10.1016/j.joca.2016.03.004 (PMC4967443; doi:10.1016/j.joca.2016.03.004)
Supplement: Supplementary file 3 [file mmc3.docx]

Supplementary file 3: Summary of studies including study design and scoring system for US-detected pathology

|  |  | **Scoring system** | | | **US assessment characteristics** | |
| --- | --- | --- | --- | --- | --- | --- |
| Author, year | **Study design** | **Effusion** | **Synovial hypertrophy** | **Doppler signal** | **Suprapatellar area** | **Knee position** |
| Beitinger, 2013 | Case-control | - | - | Colour Doppler (summative score 0-12 from supra-, infra-patellar, medial and lateral scan (grades 0-3 for each)) | - | - |
| Bevers, 2014 | Cross-sectional | ≥4mm | ≥2mm | - | Midline | Neutral |
| Chan, 2014 | Cross-sectional | ≥4mm | ≥4mm | - | - | - |
| Chatzopoloulos, 2008 | Case-control | ≥2mm  (absent <2mm, small 2-5 mm, large >5mm) | - | - | - | 30-40^o^ of flexion |
| D'Agostino, 2005 | Cross-sectional | ≥4mm | ≥4mm | - | Midline | 45^o^ of flexion |
| Hall, 2014 | Case-control | ≥4mm | ≥4mm | Power Doppler (grades 0-3: normal/absent, mild, moderate, marked/severe) | Multi-planar | 30^o^ of flexion |
| Jung, 2006 | Case-control | ≥2mm | - | - | - | Neutral |
| Kumm, 2009 | Cross-sectional | ≥4mm | ≥4mm | Doppler (presence/absence) | - | - |
| Mendieta 2006 | Cross-sectional | ≥2mm | - | - | Midline | 30^o^ of flexion |
| Naredo, 2005 | Case-control | ≥4mm | - | - | - | 30^o^ of flexion |
| Picerno, 2013 | Cross-sectional | ≥2mm | - | - | Multi-planar | Neutral |
| Song, 2009 | Case-control | ≥2mm (grades 0-3: normal (< 5 mm), slight (5-7 mm), moderate (8-10 mm), strong (≥11 mm)) | ≥4mm (grades 0-3: normal (0mm), slight (>0 to <4 mm), moderate (4-7 mm), strong (≥8 mm)) | Power Doppler (grades 0-3: normal/absent, mild, moderate, marked/severe) | Multi-planar | Less than 90^o^ of flexion |
| Tarhan, 2003 | Case-control | ≥2mm (grades 0-3: absent (<2 mm), mild (2-4 mm), medium (5-10 mm), severe (>10 mm) | ≥2mm (grades 0-3: absent (<2 mm), mild (2-5 mm), medium (6-8 mm), severe (>8 mm) | - | Multi-planar | Neutral |
| Tchetina, 2013 | Case-control |  | ≥4mm | - | - | - |
| Ulasli, 2014 | Cross-sectional | ≥4mm (grades 0-3: grade 0 (< 4 mm), grade 1 (4-8 mm), grade 2 (8-10 mm), grade 3 (≥11 mm)) | - | - | Multi-planar | 30^o^ of flexion |
| Walther, 2001 | Case-control | - | - | Power Doppler (grades 0-3: normal/absent, mild, moderate, marked/severe) | Multi-planar | - |
| Wu, 2012 | Case-control | ≥4mm (grades 0-3: grade 0 (< 4 mm), grade 1 (4-8 mm), grade 2 (8-10 mm), grade 3 (≥11 mm)) | ≥4mm (grades 0-3: grade 0 (< 4 mm), grade 1 (4-8 mm), grade 2 (8-10 mm), grade 3 (≥11 mm)) | - | Multi-planar | - |
| Zivanovic, 2009 | Case-control | ≥4mm | ≥4mm |  | - | - |
| Kristoffersen, 2006 | Case-control | Absent/present | Absent/present | Colour Doppler  (absent/present) | Multi-planar | - |
| Arthul, 2014 | Cross-sectional | Absent/present | - | - | - | - |
| Blankstein, 2006 | Case-control | Absent/present | Absent/present |  | Multi-planar | - |
| Iagnocco, 2014 | Cross-sectional | Absent/present (grades 0-3: normal, mild, moderate, marked/severe) | Absent/present (grades 0-3: normal, mild, moderate, marked/severe) | Power Doppler (grades 0-3: normal/absent, mild, moderate, marked/severe) | - | - |
| Malas, 2014 | Cross-sectional | Absent/present | - | - | - | - |
| Svetlova, 2010 | Case-control | Absent/present (grades 0-3: absent (0 mm), mild (≤3 mm), medium (4-6 mm), severe (>6 mm) | - | - | Multi-planar | - |
| Abraham, 2014 | Cross-sectional | ≥4mm | - | - | Multi-planar | 30^o^ of flexion |
| D'Agostino, 2015 | Cross-sectional | Absent/present (grades 0-3) | Absent/present (grades 0-3) | Absent/present (grades 0-3) | Multi-planar | Different degrees of flexion |
| Martino, 1992 | Cross-sectional | - | - | - | - | Neutral |
| Mielke, 1990 | Case-control | - | - | - |  |  |
| Schmidt, 2004 | Cross-sectional | - | - | - |  |  |
