# Supplementary material for: Synovial changes detected by ultrasound in people with knee osteoarthritis – a meta-analysis of observational studies
Source: Osteoarthritis Cartilage. 2016 Aug;24(8):1376–83. doi: 10.1016/j.joca.2016.03.004 (PMC4967443; doi:10.1016/j.joca.2016.03.004)
Supplement: Supplementary file 4 [file mmc4.docx]

Supplementary file 4: Quality assessment of cross-sectional and case-control studies using the Newcastle-Ottava Scale

A: Quality assessment of cross-sectional studies using the Newcastle-Ottava Scale

| Author, year | Selection | | | | Comparability | Outcome | | Total stars | Standardised quality score (% of the maximum score) |
| --- | --- | --- | --- | --- | --- | --- | --- | --- | --- |
|  | 1 | 2 | 3 | 4 | 5 | 6 | 7 |  |  |
| Bevers, 2014 | ✯ |  |  | ✯✯ |  | ✯✯ |  | 5 | 50 |
| Chan, 2014 | ✯ |  |  | ✯✯ |  | ✯✯ | ✯ | 6 | 60 |
| D'Agostino, 2005 | ✯ | ✯ |  | ✯✯ | ✯✯ | ✯✯ | ✯ | 9 | 90 |
| Kumm, 2009 | ✯ |  |  | ✯ | ✯✯ | ✯✯ |  | 6 | 60 |
| Mendieta, 2006 |  |  |  | ✯✯ | ✯✯ | ✯✯ | ✯ | 7 | 70 |
| Picerno, 2013 | ✯ |  |  |  |  | ✯✯ |  | 3 | 30 |
| Ulasli, 2014 | ✯ |  |  | ✯✯ |  | ✯✯ | ✯ | 6 | 60 |
| Arthul, 2014 |  |  |  |  |  | ✯✯ |  | 2 | 20 |
| Iagnocco, 2010 | ✯ |  |  | ✯✯ |  | ✯✯ |  | 5 | 50 |
| Malas, 2014 |  |  |  | ✯✯ |  | ✯ | ✯ | 4 | 40 |
| Maximum number of stars per question (10) | 1 | 1 | 1 | 2 | 2 | 2 | 1 |  |  |

B: Quality assessment of case-control studies using the Newcastle-Ottava Scale

| **Author, year** | **Selection** | | | | **Comparability** | **Exposure** | | | **Total stars** | **Standardised quality score (% of the maximum score)** |
| --- | --- | --- | --- | --- | --- | --- | --- | --- | --- | --- |
|  | 1 | 2 | 3 | 4 | 5 | 6 | 7 | 8 |  |  |
| **Beitinger, 2013** | ✯ |  |  | ✯ |  | ✯ | ✯ |  | 4 | 44.44 |
| **Chatzopoloulos, 2008** | ✯ |  | - | - | - | ✯ | - |  | 2 | 22.22 |
| **Hall, 2014** | ✯ |  | ✯ | ✯ |  | ✯ | ✯ |  | 5 | 55.56 |
| **Jung, 2006** | ✯ | ✯ | - | - | - |  | - |  | 2 | 22.22 |
| **Naredo, 2005** | ✯ | ✯ |  | ✯ |  | ✯ | ✯ |  | 5 | 55.56 |
| **Song, 2008** | ✯ |  |  |  |  | ✯ |  |  | 2 | 22.22 |
| **Tarhan, 2003** | ✯ |  |  | ✯ |  | ✯ | ✯ |  | 4 | 44.44 |
| **Tchetina, 2013** | ✯ |  | ✯ | ✯ | ✯ | ✯ |  |  | 5 | 55.56 |
| **Walther, 2001** | ✯ |  |  | ✯ |  | ✯ | ✯ |  | 4 | 44.44 |
| **Wu, 2012** | ✯ |  |  | ✯ | ✯✯ | ✯ | ✯ |  | 6 | 66.67 |
| **Zivanovic, 2009** | ✯ |  | - | - | - |  | - |  | 1 | 11.11 |
| **Kristoffersen, 2006** | ✯ | ✯ |  |  |  | ✯ | ✯ |  | 4 | 44.44 |
| **Blankstein, 2006** |  |  |  | ✯ | ✯✯ |  | ✯ |  | 4 | 44.44 |
| **Svetlova, 2010** | ✯ |  |  | ✯ | ✯✯ |  | ✯ |  | 6 | 66.67 |
| **Maximum number of stars per question (9)** | 1 | 1 | 1 | 1 | 2 | 1 | 1 | 1 |  |  |
